# Modelling the economic constraints and consequences of anaesthesia associate expansion in the UK National Health Service: a narrative review

**DOI:** 10.1016/j.bja.2024.01.015

**Published:** 2024-02-09

**Authors:** Stuart B. Hanmer, Mitchell H. Tsai, Daniel M. Sherrer, Jaideep J. Pandit

**Affiliations:** 1 Department of Anaesthesia, University Hospitals Coventry and Warwickshire NHS Trust, Coventry, UK; 2 Department of Anesthesiology, Orthopedics and Rehabilitation, and Surgery, Larner College of Medicine, University of Vermont, Burlington, VT, USA; 3 Department of Anesthesiology, University of Colorado School of Medicine, Aurora, CO, USA; 4 Department of Anesthesiology and Perioperative Medicine, University of Alabama Birmingham, Birmingham, AL, USA; 5 Nuffield Department of Anaesthetics, Oxford University Hospitals NHS Foundation Trust, Oxford, UK; 6 Nuffield Department of Clinical Neurosciences, University of Oxford, Oxford, UK

**Keywords:** health economics, medical education, medical staffing, operating theatres, patient safety, service delivery, workforce

## Abstract

Shortages in the physician anaesthesia workforce have led to proposals to introduce new staff groups, notably in the UK National Health Service (NHS) Anaesthesia Associates (AAs) who have shorter training periods than doctors and could potentially contribute to workflow efficiencies in several ways. We analysed the economic viability of the most efficient staffing model, previously endorsed by both the UK Royal College of Anaesthetists and the Association of Anaesthetists, wherein one physician supervises two AAs across two operating lists (1:2 model). For this model to be economically rational (something which neither national organisation considered), the employment cost of the two AAs should be equal to or less than that of a single supervisor physician (i.e. AAs should be paid <50% of the supervisor's salary). As the supervisor can be an autonomous specialty and specialist (SAS) doctor, this sets the economically viable AA salary envelope at less than £40,000 per year. However, we report that actual advertised AA salaries greatly exceed this, with even student AAs paid up to £48,472. Economically, one way to justify such salaries is for AAs to become autonomous such that they eventually replace SAS doctors at a lower cost. We discuss some other options that might increase AA productivity to justify these salaries (e.g. ≥1:3 staffing ratios), but the medico-political consequences of each of them are also profound. Alternatively, the AA programme should be terminated as economically nonviable. These results have implications for any country seeking to introduce new models of working in anaesthesia.

**Editor's key points**

• Anaesthesia Associates (AAs) are an expanding provider type, but their economic value is unknown.
• The authors consider potential AA productivity and theatre workflow and conclude that a salary of less than 50% of their medically qualified supervisors is justified economically. They note that AA salaries exceed this.
• The authors argue that AAs, as implemented, are not economically viable, and that to achieve economic viability, one of the following is required: (i) AAs need to be paid less, (ii) their autonomy should be increased, or (iii) the AA programme should be terminated.

The COVID-19 pandemic has brought into even sharper focus the anaesthetic workforce shortfall that existed before 2020. Following a commissioned report into the UK anaesthetic workforce, the Royal College of Anaesthetists (RCoA) highlighted a 1400 shortage in consultant anaesthetist posts, predicted to increase to 11,000 by 2040.^1^^,^^2^ The alarming number of more than 7 million patients now waiting for surgery^3^ adds considerably to these workforce requirements in the UK,^4^ especially as workforce shortages can also exacerbate cancellations on the day of surgery.^5^

Even before these latest challenges, alternative ways of distributing the workload using new staff groups were being considered as cost-saving measures. Historically, the UK model of working in operating theatres is not just consultant-led but in practice consultant-delivered. The contribution of trainees to elective service delivery is now negligible.^6^ Thus, the RCoA workforce calculations assume a 1:1 ratio of consultants to operating lists. This is notwithstanding the specialty and specialist (SAS) grade, unique to the UK National Health Service (NHS) who, while not being consultants, can be autonomous practitioners.^7^ The needed recruitment to these senior doctor grades to maintain the 1:1 staffing ratio is slow, given long training periods, and also expensive.

To meet this challenge a new grade of staff was conceived, Anaesthesia Associates (AAs). These can assist consultants in theatre (e.g. increase productivity by improving turnover, recovery, and discharge), or facilitate new ways of working in which there could theoretically be, say, a 1:2 supervision model, where one consultant supervises two AAs, across two theatres.

The qualifications and training of AAs are discussed elsewhere.^8^^,^^9^ Introduced to the NHS in 2004 under the original title of ‘physicians’ assistant (anaesthesia)’ or PA(A), the commitment to AA expansion is now explicit in the 2023 ‘NHS Long Term Workforce Plan’, in which NHS England and the UK Government propose to increase the number of AA training places from a 2022 baseline of 120–250 by 2028/9, and a total of 2000 by 2036/7.^10^ The AA curriculum consists of a dedicated 2-yr postgraduate diploma or master's level programme, on top of any previous qualifications (e.g. nursing or Bachelor's degree) they may have.^11^ In contrast, a postgraduate Anaesthetist in Training (AiT) will undertake a minimum total of 7–8 yr of anaesthesia/perioperative care training, in addition to a 2-yr ‘foundation programme’ undertaken by all UK medical graduates, and after a medical school course that could be up to 6 yr, making up to 16 yr in total from leaving school and not counting any higher research degrees or specialty fellowships. The shorter AA training pathway towards supervised practice could be considered a more cost-effective means of expanding the UK anaesthetics workforce. However, this is with the self-evident caveat of reduced exposure to a wider range of medical, surgical, and other specialties.

Upon graduation, AAs are encouraged to register with the Managed Voluntary Register held by the RCoA.^8^^,^^11^ Although the General Medical Council (GMC) is the UK regulatory body for doctors, it is hoped that AAs will be regulated by this same body by late 2024.^12^

As AAs are not doctors, or nurses or operating department practitioners (ODPs), how to optimise teamwork with this entirely new staff group warrants consideration. These issues relate to how best to transition from one model of working to another. The human factors aspects of this have, to a large extent, been discussed elsewhere.^13^, ^14^, ^15^, ^16^ The purpose of our review here is different: to offer an economic analysis of the AA programme. We discuss, among other things, the size of the workforce challenge, the different options available for new ways of working, and their economic consequences. Throughout this review, we use the notion of something being ‘economically justifiable’ or not. This is the point at which the innovation achieves the same output at a lower, or at least the same cost. Or if it increases cost, then productivity should at least increase commensurately. In this framework, adding the cost of AAs to the workforce should either be justified by a parallel increase in productivity (e.g. cases completed), or at least cost-neutrality by saving on costs of employing other (e.g. consultant or SAS) staff. Note that such issues are relevant to any system in transition. For systems or countries that have already long transitioned to 1:2 staffing ratios, they are less relevant—although the analysis presented here may be an opportunity for them to review the cost-effectiveness of the models as they exist in those countries.

## Estimating the size of the anaesthetic workforce shortfall in the UK

Any identified shortage of anaesthetists translates into the economic challenge of employing them. The ‘top–down’ methods used by the RCoA are not published in detail but, in principle, include the following considerations. The existing consultant (and separately, SAS) numbers are known at a point in time (from the RCoA, Association of Anaesthetists, GMC, and British Medical Association databases). A review is undertaken of advertised consultant posts. An estimate is made of patient demography, population growth, and past workforce requirements. Calculations used to inform the number of training posts are also taken into account (these numbers being themselves reliant upon estimates of future consultant requirements). Thus, the RCoA estimates around 8000 consultants and about 2000 SAS doctors (a total of ∼10,000 senior grades) currently in post, making a total of about 1400 senior doctor posts unfilled (∼1000 and ∼400 consultants and SAS posts, respectively).^17^

An alternative, ‘bottom-up’ modelling approach reaches similar conclusions about the size of the shortfall.^4^^,^^18^ We will not repeat the analysis, but the starting point is the number of operating lists to be covered. For each theatre scheduled for elective activity for 5 working days, and factoring in clinical *versus* non-clinical time in NHS contracts^19^ and for leave and other absences, the modelling predicts about three consultants needed per operating theatre. This ratio is lower if trainees contribute significantly to service (e.g. in 2010 work patterns, the modelling estimated ∼2.2 consultants per theatre^18^). Explained simply, this multiplier of three arises because two consultants are needed to cover a list for 5 days of the week, as a result of their non-clinical time, non-theatre, and on-call commitments. A third consultant is then needed to cross-cover leave. It is estimated that there are ∼4000 operating theatres in hospitals across the UK (notwithstanding the definition of ‘operating theatres’, given an increasing number of locations to which anaesthetists are assigned to deliver care).^4^ This means that by this bottom–up model ∼12,000 consultants/SAS doctors are needed in total.^4^ If there are ∼10,000 consultants/SAS doctors as estimated by RCoA surveys, this suggests a current shortfall of about 2000, which is similar to the aforementioned top–down estimate.

All these approaches, top–down and bottom–up, assume a ‘two-sessional day’ (i.e. 8 h of theatre time in contractual terms) over 5 weekdays of work. Any theatre schedules that extend the list duration to more than this (e.g. three sessions) or include planned weekend working will add to the workforce demands. However, several sources of information indicate that weekend elective work constitutes just ∼5% of total surgical activity, so any error in the bottom–up modelling estimate is modest.^20^^,^^21^

## How anaesthesia associates could help the UK workforce challenge: from assistance to 1:2 models

Many potential roles for AAs have been outlined by the national ‘Getting It Right First Time’ programme.^22^ Expanding on these themes: AAs might undertake preoperative assessments, either in a dedicated clinic in advance of surgery (thus extending the concept of nurse-led pre-assessment clinics^23^) or preparing in-patients or day-case admissions. They could place regional anaesthesia and neuraxial blocks—either in block rooms or anaesthetic rooms—in advance of surgery, or assist turnover of patients by extubating the previous patient or transferring them to recovery while the consultant prepares or induces the next. They might offer rest breaks or relief to consultants; this relief extends to facilitate consultant attendance at meetings or teaching sessions. There are potential roles for AAs in locations where currently sedation is administered by nurses, with the potential to offer deep sedation given their extended training. AAs may help departments comply with the European Working Time Directive (EWTD) for trainees and substantive staff, provide continuity within a department, and allow more rapid ‘on-boarding’ of new staff and provide a pathway for theatre staff to progress in their career development.^24^ All these activities contribute to productivity, even if the result cannot be quantified in every case. Outside of the operating theatre, AAs could provide service to obstetric anaesthesia and intensive care units.

Specifically recommended in an RCoA's document, endorsed also by the Association of Anaesthetists, is to introduce a 1:2 model of work, wherein one physician supervisor oversees two AAs across two theatres.^25^ Common practice in many other countries, then only half the number of senior staff as estimated by the aforementioned staffing shortfall calculations will theoretically be needed, with potential cost savings.

**Fig 1:**
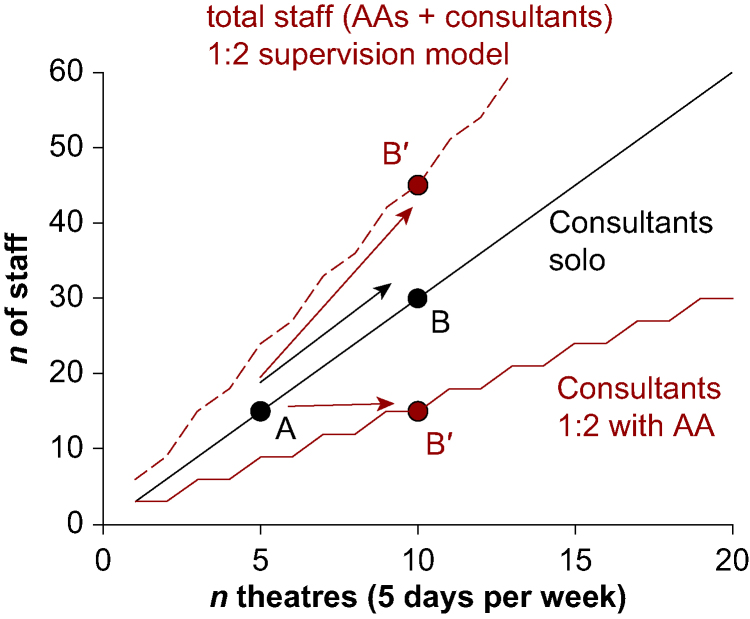
Staffing numbers needed per operating theatre (allocated two sessions over 5 days of the week). The black line represents the number of consultants needed in a 1:1 model as estimated using bottom–up methods.^18^ The solid red line represents the number of consultants needed on a 1:2 staffing model with Anaesthesia Associates (AAs; note the ‘staircase’ effect). The dashed red line represents the total staffing requirements on this 1:2 model (consultants plus AAs). For the extra staff to be cost-neutral, the cost of employing the total staff (dashed red line) at any given point on the x-axis must equal that of the solid black line at the same point. Thus, a theoretical hospital with 10 theatres but with a (greatly exaggerated) shortage of consultant workforce may lie at point A. It has a choice of either recruiting more consultants to cover all theatres (to move to point B), or instead recruiting to AA posts (points B′). Economically, the cost to the hospital of the staffing combinations at points A and B′ should be at least be equal (or the cost of B′ should be lower than B). See text and Supplementary material S1 for further details.

Figure 1 shows from the perspective of a single hospital how the number of consultants needed rises with the number of operating lists to be staffed, for traditional 1:1 (black line) and then 1:2 (red lines) models of supervision (details of how this is calculated are provided in the Supplementary material S1). For the given hospital, these lines translate into salary costs (something which the Royal College document failed to consider^25^). There are two important implications of Fig. 1. One is that there are more staff in total under any 1:2 supervision model: UK consultants will need to become skilled in supervising more than one theatre, as a new way of working, and this will have human factors and team-building implications. A second is that when transitioning into a 1:2 model to be cost-neutral, the total cost to the hospital of employing the extra staff (dashed red line) must equal the current cost of the 1:1 model (the solid black line), at any given point on the X-axis. We therefore also need to consider how much AA supervisees could maximally be paid, as a proportion of their supervisor's salary. We discuss these two consequences, team and economic, in turn.

## Team implications of the 1:2 model

UK consultants are generally used to managing anaesthesia trainees who require supervision and teaching. These trainees are destined to become future colleagues of those consultants. There has never been the preparation of UK consultants in the supervision of those who require no teaching or training.^26^ Anaesthetic trainees might be described as in a ‘dynamic career state’, their responsibilities and capabilities increasing with time. In contrast, AAs are ‘static’ in the sense they will never progress in any career hierarchy as currently defined. Arguably, AAs are akin to SAS doctors without autonomous practice status and who require constant consultant supervision.^7^ Moving to supervise this heterogenous group of professionals—anaesthetic trainees, non-autonomous SAS doctors, and AAs, along with working with anaesthetic nurses and ODPs as part of the team—will necessitate changes in the work dynamic and the training and preparation of consultants.^26^

There may be an adverse impact on AiT training if consultants are instead supervising AAs, whether in a 1:1 or a 1:2 model. Although a specific definition of a ‘training list’ may vary with context (e.g. the grade of the trainee, the type of list, and training objectives), generally these are deemed to be those where a trainee is assigned as supernumerary to a consultant on a list. More distant supervision is denoted as a ‘2B’ supervision model; ‘a supervisor is within the hospital and available for queries and able to provide prompt direction or assistance’.^27^ Specifically, a model in which a single consultant supervises two theatres, one staffed by an SAS doctor (or AA) and one by a physician trainee, is not deemed to be a ‘training list’ for the purposes of trainee accreditation. This is because, for completion of training milestones, dedicated ‘Assessment Faculty’ (as defined by the RCoA as including consultants or SAS grade doctors) must provide feedback for the AiT.^28^ Direct observation is necessary, and this cannot be achieved within a 1:2 supervision model.

In their survey of anaesthetists in training, Evans and colleagues^9^ found that a third of respondents with prior experience of working with AAs reported a negative impact on their training, with a main concern being that although AAs are not required to complete any further postgraduate examinations, they, in essence, consume consultant-supervision hours. An additional concern raised in this survey was that the scope of practice for AAs needed a sharper definition, including whether AAs were—or should be—in a position to supervise physician trainees (or *vice versa*). Following a ‘grassroots’ campaign, the ‘Anaesthetists United’ group has been formed, making use of social media to canvass support and successfully call for an extraordinary general meeting (EGM) of the RCoA^29^, ^30^, ^31^ on October 17, 2023. Of the six motions proposed and carried in the EGM, three directly addressed concerns related to recruitment and supervision of AAs: (i) requesting a pause on AA recruitment until a scoping study of their impact on AiTs was completed; (ii) ensuring that AiTs are given priority for training opportunities over AAs; and (iii) more clarity with regard to who should supervise whom. Other motions related to the need to ensure direct consultant supervision whenever an AA works outside of their initial scope of practice, and ensuring patients are informed specifically that the AA is not a doctor.^29^^,^^32^ Structured interviews have shown, both in the UK^33^ and in the USA^34^ that patient satisfaction is increased by greater clarity and information about the qualifications of non-physician roles. These concerns reflect the potential beginnings of tensions described by US authors in relation to professional disputes and could represent an early, but real risk in an already fraught dialogue within the profession.^35^^,^^36^

## Lessons from the USA experience

In the USA, Certified Registered Nurse Anesthetists (CRNAs) are long established, but the evolution of their practice has resulted in significant tension between the American Society of Anesthesiologists (ASA) and the American Association of Nurse Anesthesiology (AANA).^37^ To date, 22 states have opted out of the requirement for physician supervision of CRNAs, following the 2001 amendment to the Medicare and Medicaid Anesthesia Services Conditions of Participation. There are now several ongoing, expensive legal cases between the ASA and AANA over CRNA autonomous practice.^38^

Although it has long been recognised that CRNAs might assist with workflows as described earlier for AAs, the key debates now centre around whether they should work autonomously or only under physician supervision. This has also extended into pay disputes; the AANA's argument is that if autonomous, then CRNAs should logically be paid the same as physician anaesthesiologists.^13^^,^^39^

Studies examining the quality impact of CRNA autonomous practice have been contradictory. Some studies have concluded that anaesthesiologists deliver safer patient care,^40^ with increased mortality rate in non-anaesthesiologist-directed care.^41^ Some contrary studies indicate no impact on patient outcomes with autonomous CRNA-delivered care.^42^^,^^43^ The difficulty in adjusting for patient factors in these studies makes it almost impossible to ascertain the true outcome.^44^^,^^45^

As part of a medical workforce recovery programme following the COVID-19 pandemic, the American Medical Association established the ‘Fighting Scope Creep’ campaign^46^ to advocate physician-led care to improve patient safety. This includes support for court cases objecting to the expansion of non-physician health care workers, including CRNAs. Indeed, 2021 saw the AANA rebrand itself as the ‘American Association of Nurse Anesthesiologists’. (Note: In the UK, parallels can be drawn between the ‘Association of Anaesthetists’, a physician organisation *vs* the ‘Anaesthesia Associates’, the name for AAs.) This sparked backlash from the ASA who claimed this was ‘misappropriation of title’.^47^ The New Hampshire Supreme Court upheld a ruling from the New Hampshire Board of Medicine that the term ‘anesthesiologist’ is restricted to use by licensed physicians in that state.^48^

Such issues are an extreme form of some of the anxieties implied by the motions at the RCoA EGM (discussed earlier). They could translate into challenging team dynamics within the operating theatre. Hence, even the most carefully planned teamwork, honed by joint training within an organisation, may be disrupted by forces outside the organisation's or team's control. Although some US anaesthesia care teams have managed to overcome the scope of practice tensions, it has not come without a significant investment of time and resources.^49^^,^^50^

## Economics of the 1:2 model: setting the envelope for maximum viable Anaesthesia Associate salary

We now turn to the financial modelling. There is no economic value to introducing AAs as an end in itself; economic value arises only from the resulting patterns of work yielding cost savings, or delivering service at lower cost. Figure 1 helps estimate the maximum salaries that AAs could receive to justify their introduction. The black line for the total number of consultants translates into the current financial envelope on the traditional 1:1 model of service delivery. Imagining, therefore, the simplest case of a day across two operating theatres, currently staffed by two consultants, it is self-evident that in any new 1:2 model, the salary of the two new supervised AAs cannot exceed in total the salary of the single supervisor they replace. Therefore, crudely speaking, an AA cannot rationally be paid more than 50% of their supervisor's salary.

**Fig 2:**
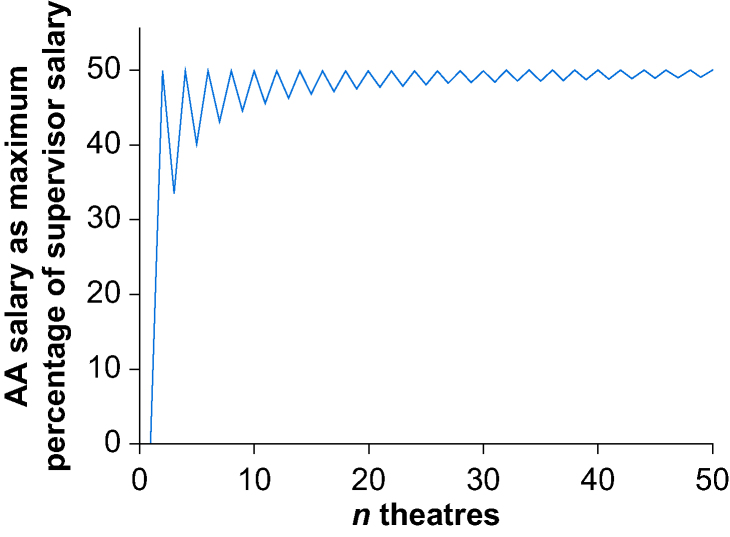
The maximum economically viable salary that can be awarded to an Anaesthesia Associate (AA), as a proportion of their supervisor's salary, assuming a 1:2 supervision ratio per operating theatre (allocated two sessions over 5 days of the week), and based on Fig. 1. The ‘sawtooth’ pattern arises as a result of the ‘staircase’ relationship in Fig. 1, itself a function of the absences because of leave and nonclinical time within contracts. This relationship assumes that AAs have the same leave and nonclinical time entitlements as their supervisors (see Supplementary material S1 for further details).

More accurately, this estimate needs to take into account the total number of staff needed across a suite of theatres, and not just the simplest-case consideration of two theatres. Figure 1 also indicates the total number of staff needed to cover a suite of *n* theatres in the 1:2 model (dashed red line). The cost savings in replacing one consultant with two AAs across *n* theatres can then be calculated, and Fig. 2 expresses this as the ‘maximum % of supervisor salary receivable by an AA to maintain cost neutrality’. There is a sawtooth relationship (as a result of the staircase effect seen in Fig. 1) but the estimate asymptotes to 50%, as predicted by the simplest-case example of *n*=2 theatres discussed earlier. In fact, for very low numbers of theatres, the maximum salary is much less than 50%. This is explained (*reductio ad absurdum*) by the fact that there is no advantage in adopting an AA model where there is a single operating theatre (*n*=1) and so the maximum economically rational salary for an AA in this extreme situation of one operating theatre is technically zero.

Figure 2 does not require knowledge of the actual salaries in currency because it is described only as proportions (a ‘ratiometric analysis’). In converting to currency, it may be instinctive to assume that the supervisor is a consultant, whose published salary scale ranges from £93,666 to £126,281 (not counting additional programmed activities, on-call supplements, and clinical impact awards).^51^ This means the economically viable salary for AAs should range from £46,833 to £63,140. However, autonomous SAS doctors can in fact be AA supervisors and their salaries range from a more modest £58,756 to £80,000, meaning that in fact, the true economically viable salaries for AAs cannot be more than within the range of £29,378–£40,000. In other words, the reference point for planning AA employment contracts and salaries is logically the lowest level of supervisor salary (the ‘margin’). Otherwise, there is no economic justification for creating a new grade of staff (AAs) who actually cost more because they require supervision.

Given these economic realities, the present situation raises several concerns. Firstly, we discovered that AA salaries vary greatly by region. In contrast to doctors or nurses whose pay is set nationally across the NHS, this suggests local staffing pressures are dictating AA salaries.

**Fig 3:**
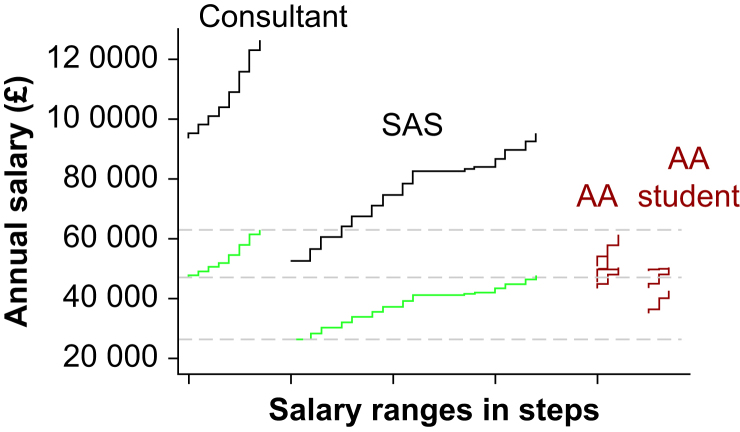
Salary ranges for different staff grades. The black lines show the published salary steps for consultants and specialty and specialist (SAS) doctors, respectively, with the green lines the corresponding 50% values. The dashed horizontal grey lines are, from top to bottom, the 50% thresholds of the highest basic consultant salary, of the lowest basic consultant salary (which also corresponds to the 50% value of the highest SAS salary), and of the lowest basic SAS salary, respectively. The first group of red lines are from published adverts for qualified Anaesthesia Associates (AAs) (see text) from three Trusts, including one for an Assistant Professor AA. The last group of red lines is from published adverts for student AAs (see text).

Secondly, and perhaps arising from the first, absolute salaries are high. Figure 3 summarises these salary relationships. Although NHS Health Careers suggests that a qualified AA should be paid ‘ … at least band 7 with the chance for additional career progression’^52^ equating to £43,742–£50,056,^53^ several Trusts have advertised positions for both student and qualified AAs in excess of this. Thus, the University College London Hospitals NHS Foundation Trust is offering a Band 8a salary (£58,698–£65,095) to qualified AAs.^54^

Thirdly, even student AA salaries are high. One Trust has offered students a Band 6 salary of £35,392–£42,618.^55^ In its 2022/23 financial year funding guide, Health Education England advised that it will provide funding for up to 120 student AAs, with a salary range of £46,402–£48,472.^56^ NHS Scotland has advertised trainee AA posts on Band 7 (Fig. 3).^57^ Compare these levels of salary support with medical students who need to take loans to study and now qualify with average debts >£100,000.^58^

Fourthly, the ‘static’ concept of AA careers is changing with one university advertising a professor position to an AA (Assistant Professor, Anaesthesia Associate Education) at the salary range of £45,585–£61,198.^59^ All these salaries cited are plotted in Fig. 3. It is clear that actual AA salaries (including those advertised for students with no clinical commitments) not only exceed the calculated economically rational threshold, but also are actually higher than many salary steps within the SAS grade. It is difficult to justify any of this from a purely economic perspective.

## Economics of other models of Anaesthesia Associate working

The aforementioned argument has assumed that a 1:2 model is as productive as the 1:1 model of a consultant assigned to each theatre. This is a generous (to the AA model) assumption because the AA assigned to a theatre will likely not be as productive as the consultant they have replaced. However, balanced against this is a potential consultant contribution to improve patient turnover in the 1:2 model (i.e. the consultant improves productivity by helping turnover across the two AA-staffed theatres they supervise).

We have also assumed that a 1:2 supervision model is indeed more productive than the other ways that AAs can contribute to service delivery. In models other than 1:2, AAs could maximise case numbers completed by working across several consultant-staffed theatres to reduce or eliminate turnover time (the gap time between successive patients on a list). This could arise by them undertaking blocks or neuraxial anaesthesia, or taking previous patients to recovery while the next is induced, or offering breaks and relief and maintaining energy and morale. There are several counterarguments suggesting that this is not more economically productive than the 1:2 model.

One is that the potential gains achievable by eliminating turnover times are often greatly exaggerated. The NHS England Model Health System, incorporating the Model Hospital,^60^ monitors UK hospital performance and uses a measure of utilisation (capped utilisation) that purportedly reflects the amount of time that is wasted and therefore offers potential for productivity gains. However, this measure has been criticised for using ‘false accounting methods’ in analysis.^61^ Even if regarded as valid, the potential gains are, using the NHS’ own (albeit exaggerated) methods, limited to just ∼15% of the total list time.^62^^,^^63^ Therefore, eliminating turnover time does not economically justify any AA salary >15% of their supervisor's salary, assuming a single AA is assigned to a single consultant-staffed list. Expressed another way, the activity of the AA can be viewed as replacing (saving) a consultant's salary, or enhancing the output of a single consultant on a list. Therefore, it is the percentage of consultant workload enhanced—or replaced—that represents the economic justification of AA salary. By this calculation, an AA would reliably need to cross-cover at least four consultant-staffed theatres in a time-aligned manner to eliminate enough turnover time (i.e. a maximum ∼15% per list, multiplied by four) such as to economically justify a salary of ∼50% of a consultant grade. Even if this were possible, this would result in overall fewer AAs than consultants; the reverse of a 1:2 staffing model.

This model of work is a form of facilitating ‘overlapping anaesthesia’. By starting procedures in the next patient while a previous one is still in theatre (such as a regional block, neuraxial anaesthesia, or invasive monitoring), this could shorten the total operating time for some specific surgeries. This was in fact the norm historically, so any economic modelling in this regard is not unique to the AA proposition; it can apply equally well to physician trainees, as it did in the past. More recent governance regulations requiring a nurse to be assigned to each anaesthetist means that any economic gains from such overlapping practice are questionable because an additional nurse would also need to be employed for any AA engaged to overlap. A more extreme form of overlapping is in so-called high-intensity lists. In this model, a single surgeon or single surgical team works across two (or more) theatres, with two (or more) anaesthetic teams working to prepare the next patient ready for surgery before the operation is completed on the previous. In this way, surgical downtime is eliminated with the appearance of significantly greater efficiency. However, modelling of the workflows for high-intensity lists has already shown that they can never be more productive than two separate theatres with their own teams,^64^ and that they are considerably more expensive.^65^ Therefore, even in this most extreme case, the use of AAs within such a working arrangement might to some extent address the latter (expense), if paid at appropriately lower rates, but will do nothing to address the former (productivity). In other words, the productivity gains from overlapping are at best modest as compared with a 1:2 model and autonomous working.

Similar conclusions have been reached in analyses of working practice in the USA. Abouleish and colleagues^66^ reported, as we do, that CRNAs are not in fact any cheaper than physicians, taking into account that the latter are autonomous. Both Abenstein and colleagues^67^ (from a physician perspective) and Cintina and colleagues^68^ (from a nursing perspective) concluded that supervised CRNAs were potentially economically viable, but only if the salaries of the latter were no more than 50% of the former.

## Options appraisal for making the high Anaesthesia Associates salaries economically viable

The aforementioned analysis has focussed on ‘salaries’ as a surrogate for ‘costs’. The former are what is received by individuals, the latter what is borne by employers or the system. Costs are higher than salaries because of elements such as pension contributions, study budgets, or other support costs. However, in the NHS these costs are always proportionate to salary; hence the utility of starting with a ratiometric analysis and then only later converting to actual currency. An even more complete analysis of costs from the system perspective might include the costs incurred in training the different types of staff (those for physicians far exceeding AAs). However, that analysis would then also need to include the element of those costs borne directly or indirectly by individuals (i.e. the student debt of junior doctors referred to earlier). Ultimately, therefore, salaries are a useful surrogate for costs—and relevant. It is not persuasive, for example, to argue that salaries for a given staff grade should be constrained because instead the investment has been spent on their training.

Given the analysis above that AA salary levels are *de facto* surprisingly high, we consider here other ways of working that might achieve economic justification for these (Supplementary material S2 and S3). The most superficially straightforward is to make AAs autonomous practitioners, unconstrained by the 1:2 supervision model. Becoming then functionally equivalent to senior doctors, any salary marginally lower than their supervisor's represents cost saving. However, as discussed above in relation to tensions in the USA, seeking autonomous practice may itself have profound and predictable medico-political consequences.

Another option is to increase the 1:2 supervision model to 1:3, or 1:4 or even higher ratio (i.e. a consultant/SAS doctor supervises three, four, or more theatres). The higher the ratio, the fewer supervising physicians that are needed, and the more each AA can then be paid with the monies saved from these senior physician salaries. Supplementary material S2 presents the detailed modelling of a 1:3 ratio. However, evidence from the USA and Europe suggests that patient safety is a concern, with more frequent adverse events in supervision ratios greater than 1:2.^69^ This is supported by stochastic simulation^70^ and retrospective record review.^71^ Given that 1:2 will be a huge leap in the UK from a 1:1 model, it is unlikely that higher ratios will be embraced quickly, if at all.

A third option is that, within the 1:2 supervision model, AAs receive fewer leave and nonclinical time entitlements and so spend a greater proportion of their time delivering clinical care. This means that fewer of them will be needed overall than the assumptions made in Fig. 1, and therefore, each individual AA can be paid more. However, this may come at the cost of fatigue, with attendant safety implications. To reach the conclusion that the economically rational AA salary is no more than 50% of their supervisor's salary, we made the assumption that AAs deliver the same quantum of clinical care in the working week as their supervisors. Supplementary material S2 shows how, if this proportion of clinical time is greatly increased then this calculated salary threshold would rise. With reduced time away from clinical contact, AAs would then be paid very low rates ‘per hour in theatre’ but their total salaries could then approximate their supervisor's because they spend many more hours in theatre. Whether this balance of activities in a job plan is sufficiently attractive to sustain the AA model is a separate question, and it is not known what the precise contractual arrangements currently apply, given that AA contracts are not standardised nationally.

## Conclusions

**Fig 4:**
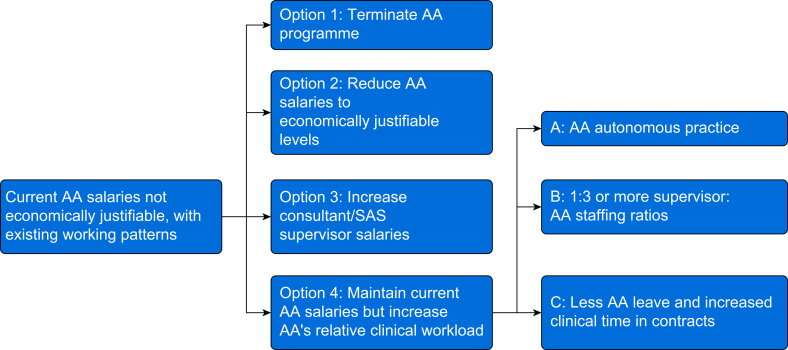
A logic or decision tree of available options for rationalising Anaesthesia Associate (AA) salaries and work patterns. The starting point is the conclusion that, under existing working arrangements, current AA salaries cannot be economically justified. The options are broadly (not in order of any preference) to terminate the AA programme; to reduce AA salaries to less than 50% of specialty and specialist (SAS) doctor grades; to increase supervisor salaries, such that current AA salaries are less than 50% of SAS doctor grades; or to maintain the current AA salary levels but increase their clinical commitments. In this last domain, there are broadly three options: implement autonomous practice for AAs, increase staffing ratios to 1:3 or more, or to adjust AA contracts to maximise delivery of clinical service. These options are not all mutually exclusive, and a combination of several may be feasible (other than termination of the programme which is a definitive option).

Introducing AAs into the UK workforce could indeed save costs and contribute meaningfully to service delivery but only if one or more options, discussed above and summarised in Fig. 4, are implemented. Even then, the new models of work within these options have implications for the preparation of consultants in a new form of teamwork and require careful planning to ensure there are no adverse effects on AiT training.

Despite the objective analysis, it is clear that AAs, including student AAs with no clinical responsibilities, are currently employed at salaries far beyond this 50% envelope. This lack of economic rationale undermines the driver that led to the creation of AAs in the first place, which was to limit or reduce costs. However, this original cost-saving objective may have been later supplanted by the now greater and urgent need to undertake the work, regardless of cost. To refer back to Fig. 1, for the understaffed hypothetical hospital sitting at point A with empty theatres, filling these with work accrues income. Directing all this income to employing AAs and waiving all profit are potentially worthwhile because more patients are treated, even if it results in greater-than-optimal AA salaries. Government planners may have concluded it is important or necessary to expand AA supply to enable hospitals to move more rapidly from point A to point B′ (Fig. 1). This is notwithstanding the fact that in the NHS, under all payment models to date, income to hospitals does not meet the actual costs of surgery even when lists are run perfectly efficiently.^72^ Nevertheless, this approach may be misguided in the long run, as high AA salaries, especially that of student AAs (Fig. 3), create cost pressures for other staff groups and for the future. At the time of writing, UK consultants and trainees are undertaking prolonged industrial action based on an argument for ‘pay restoration’ (i.e. to make up for pay lost over time through past below-inflation pay awards).^73^ In addition, SAS-grade doctors have entered talks with the UK Government to discuss similar pay issues.^74^ Future tensions could arise based on what seems to be a planned erosion of pay differentials across the different staff groups as shown in Fig. 3.

In summary, for the UK to transition in an economically viable way to the new working model there seem to be only two rational choices: (1) either maintain these high AA salaries and maximise AA productivity to justify them; or (2) reduce AA salaries to economically justifiable levels. In the first domain of (option 1), the following measures could maximise productivity: (i) grant AAs autonomous practice; (ii) adopt supervised practice in 1:3, 1:4, or more models of work; (iii) limit or curtail nonclinical time and leave absences in AA contracts. Each has its own profound consequences. The second domain of (option 2), although rational, could make AA posts so unattractive as to inhibit recruitment.

A final option is of course to recognise all these realities and limitations, and to conclude that the UK AA programme, as it has evolved, is in fact economically nonviable and to terminate it.

## Authors’ contributions

Conceived the study in the context of the UK situation: SBH, JJP

Wrote the first draft, co-wrote subsequent versions, and approved the final version: SBH

Undertook the comparative analysis of the US situation, co-wrote subsequent versions, and approved the final version: MHT

Undertook the comparative analysis of the US situation, co-wrote subsequent versions, and approved the final version: DMS

Conceived the study in the context of the UK situation: JJP, SBH

Wrote the first draft, co-wrote subsequent versions, finalised the figures, and approved the final version: JJP

## Declarations of interest

JJP is the Clinical Director, Operating Theatres Oxford University Hospitals and for the Berkshire, Oxfordshire, Buckinghamshire (BOB) Integrated Care System (ICS). MT is the President, and DS Director-At-Large, of the American Association of Anesthesia Clinical Directors. No other competing interests or funding is declared.
