# Supplementary material for: Modelling the economic constraints and consequences of anaesthesia associate expansion in the UK National Health Service: a narrative review
Source: Br J Anaesth. 2024 Feb 9;132(5):867–76. doi: 10.1016/j.bja.2024.01.015 (PMC11103085; doi:10.1016/j.bja.2024.01.015)

**Online Supplement S1**

**Estimating the total number of staff needed for a 1:2 supervision model**

The number of consultants operating solo (1:1 consultant to theatre model) is already robustly estimated as ~3 times the number of theatres, where these are allocated ‘two-session’ lists, over 5 days per week.^4^ This takes into account the various types of leave (including estimates for sick leave as based on large datasets; see below) and recognises the non-clinical time in consultant contracts. Therefore, for 3, 4, 5, 6…(n) theatres, 9, 12, 15, 18,…(3n) consultants are needed.

On a 1:2 staffing model of consultants to AAs, Figure S1.1 illustrates how the work is allocated and the numbers in Table S1.1 are derived.

**Figure S1.1**. Illustration of calculating staffing on a 1:2 model. Each box represents a theatre to which an AA is allocated. The C (Cons) represents the supervising consultant. The middle column shows the number for the set illustrated. The last column multiplies the AAs and Consultant numbers in the middle column by 3 to take account of non-theatre time in contracts and leave.


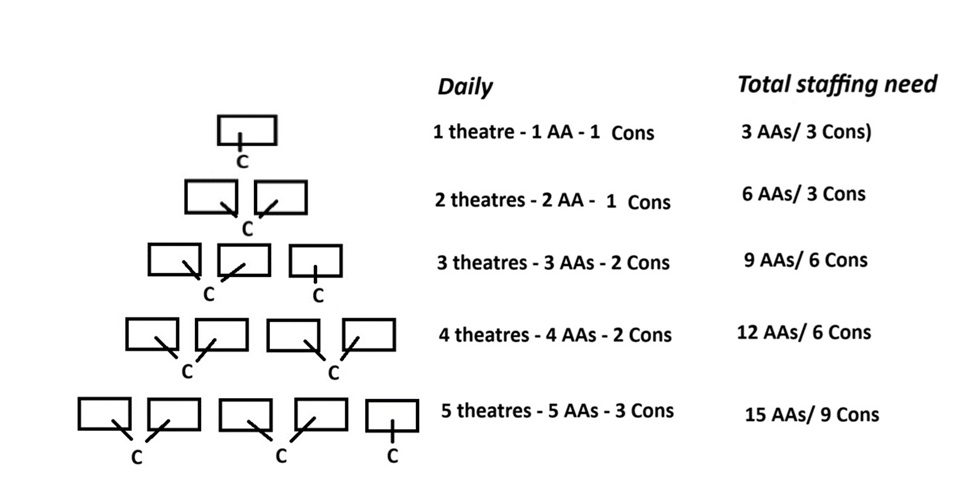


The staffing model^18^ assumed that consultants worked between 10-11 Programmed Activities (PAs) in their contract and had 25% non-clinical time (2.5 supporting professional activities) such that they delivered between 5 and 6 4-hour sessions per week when present. Absence was estimated to include annual leave (30-34 days, median 32), study leave (0-10 days, median 5), professional leave (0-10 days, median 5), sick leave (0-5 days, median 2.5), making a total annual absence from work of 30 – 59 days (median 45 days); These numbers are summarised in a partial Table S1.1, below.

**Table S1.1.** Partial table of results. The number (n) of consultants in the second column is treble the n of theatres as per previous modelling^18^ which is also equivalent to the n of AAs in a 1:2 model (column 4). For the n of consultants in a 1:2 model (column 3) is 0.5n the number of theatres (with the next even n of theatres used for odd numbers of theatres), rounded up. The total number of staff is the sum of columns 3 and 4. The last column is the maximum economically viable salary of an AA. This is calculated as the n of AAs needed (column 4) divided by the n of consultant posts theoretically saved (the difference between columns 2 and 3), expressed as a %. Note the sawtooth or oscillatory nature of the result.

| N of theatres | N of consultants needed (1:1 model) | N of consultants needed (1:2 model) | N of AAs needed (1:2 model) | Total n of staff (1:2 model) | Max economically viable salary AA (n of AAs/n consultant posts saved); % |
| --- | --- | --- | --- | --- | --- |
| 1 | 3 | 3 | 3 | 6 | 0.00 |
| 2 | 6 | 3 | 6 | 9 | 50.00 |
| 3 | 9 | 6 | 9 | 15 | 33.33 |
| 4 | 12 | 6 | 12 | 18 | 50.00 |
| 5 | 15 | 9 | 15 | 24 | 40.00 |
| 6 | 18 | 9 | 18 | 27 | 50.00 |
| 7 | 21 | 12 | 21 | 33 | 42.86 |
| 8 | 24 | 12 | 24 | 36 | 50.00 |
| 9 | 27 | 15 | 27 | 42 | 44.44 |
| 10 | 30 | 15 | 30 | 45 | 50.00 |

**Online Supplement S2**

**Theoretical analysis of maximum economically viable AA salary with greater clinical commitment**

If AAs are assumed to be absent half as much as consultants, then all of these elements in the staffing model are halved. Thus, they have non-clinical time that results in their delivering 7.5 – 8.5 4-hour sessions per week, and through an as-yet unspecified combination of different types of leave are absent for a median of 22.5 days. This will result in the multiplier of 1.5n AAs per theatre as compared with 3n for consultants. The relevant table (S2.1) is shown below.

| N of theatres | N of consultants needed (1:1 model) | N of consultants needed (1:2 model) | N of AAs needed (1:2 model) | Total n of staff (1:2 model) | Max economically viable salary AA (n of AAs/n consultant posts saved); % |
| --- | --- | --- | --- | --- | --- |
| 1 | 3 | 3 | 2 | 5 | 0 |
| 2 | 6 | 3 | 3 | 6 | 100 |
| 3 | 9 | 6 | 5 | 11 | 66.7 |
| 4 | 12 | 6 | 6 | 12 | 100 |
| 5 | 15 | 9 | 8 | 17 | 80 |
| 6 | 18 | 9 | 9 | 18 | 100 |
| 7 | 21 | 12 | 11 | 23 | 85.7 |
| 8 | 24 | 12 | 12 | 24 | 100 |
| 9 | 27 | 15 | 14 | 29 | 88.9 |
| 10 | 30 | 15 | 15 | 30 | 100 |

**Table S2.1**. Partial table of results. The number (n) of consultants in the second column is treble the n of theatres as per previous modelling.^18^ For the n of consultants in a 1:2 model (column 3) is 0.5n the number of theatres (with the next even n of theatres used for odd numbers of theatres), rounded up. The number of AAs in column 4, in contrast to Table S1, is now 1.5n the number of theatres. The total number of staff is the sum of columns 3 and 4. The last column is the maximum economically viable salary of an AA. This is calculated as the n of AAs needed (column 4) divided by the n of consultant posts theoretically saved (the difference between columns 2 and 3), expressed as a %. Note the sawtooth or oscillatory nature of the result and how it asymptotes to 100%.

The staffing can be shown by modification to Figure 1 in the main paper as Figure S2.1 below.

**Figure S2.1**. Figure 1 of the main paper reproduced, with additional data. Staffing numbers needed per operating theatre (allocated 2 sessions over 5 days of the week). The black line represents the number of consultants needed in a 1:1 model.^18^ The solid red line represents the number of consultants needed on a 1:2 staffing model with AAs (note the ‘staircase’ effect). The upper dashed red line represents the total staffing requirements on a 1:2 model, where AAs have the same leave and non-clinical time as consultants. The lower dashed red line is assuming where the AAs have significantly less leave and non-clinical time. For the extra staff to be cost-neutral, the cost of employing the total staff (dashed red line) must equal that of the solid black line.


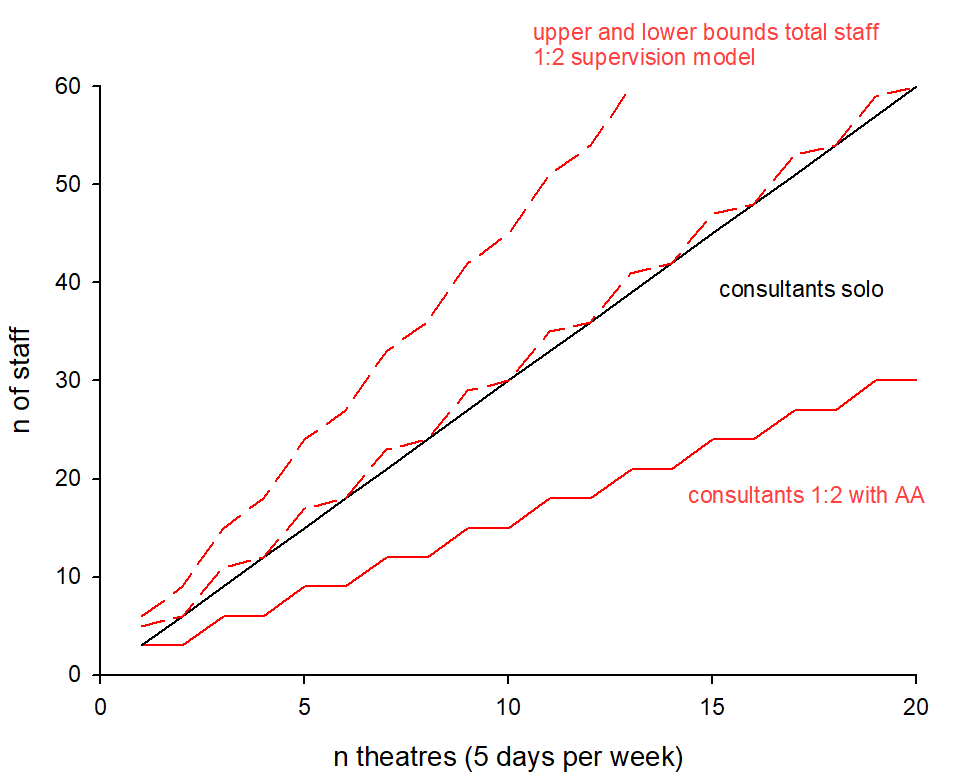


The corresponding maximum economically viable AA salary is now shown in Figure S2.2. With the reduced leave and non-clinical time commitment, this now asymptotes to 100% of consultant salary. This means that, where AAs undertake sufficiently arduous clinical workload then even under a 1:2 supervision model, the total number of extra AAs needed is sufficiently low as to justify paying the same wage as the consultant salary saved. In essence this can be understood by considering that consultants have a significant amount of non-theatre time (made up by supporting professional activity, non-theatre commitments, and leave). If an alternative provider dispenses with these, then fewer AAs are needed overall, but work harder. The analysis suggests that the point of parity is reached if AAs have overall half the leave/non-clinical entitlement as consultants. This does not imply that this situation should occur, but simply that this is the economically viable point under this condition.

In other words, it is theoretically possible to structure AA contracts in a way that justifies their higher salaries, so long as their clinical time is maximised during the working week and over the year.

**Figure S2.2.** The maximum salary that can be awarded to an AA, as a proportion of their supervisor’s salary, assuming a 1:2 supervision ratio per operating theatre (allocated 2 sessions over 5 days of the week). The ‘sawtooth’ pattern arises as a result of the ‘staircase’ relationship in Figure 1, itself a function of the absences due to leave and non-clinical time within contracts. The black line (reproduced from Figure 2 in the main paper) assumes that AAs have the same leave and non-clinical time entitlements as their supervisors. The red line assumes that they have half this entitlement.


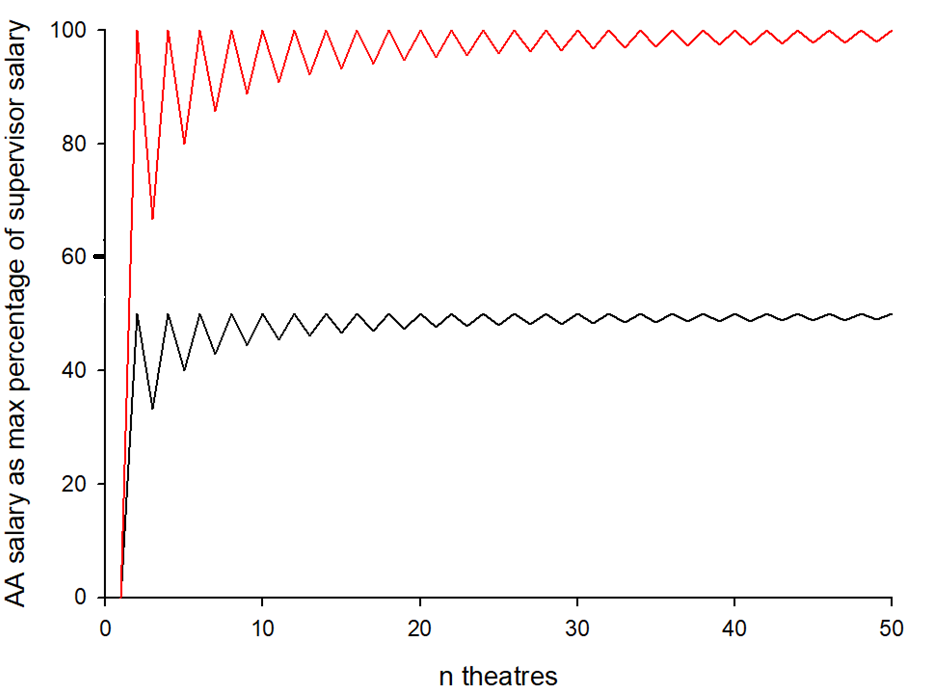


**Online Supplement S3**

**Estimating the total number of staff needed for a 1:3 supervision model**

This estimate can be derived using a similar approach to Online Supplement S1, for a 1:3 staffing model of consultants to AAs. Figure S3.1 illustrates how the work is allocated and the numbers in Table S1.1 are derived.

| N of theatres | N of consultants needed (1:1 model) | N of consultants needed (1:3 model) | N of AAs needed (1:3 model) | Total n of staff (1:3 model) | Max economically viable salary AA (n of AAs/n consultant posts saved); % |
| --- | --- | --- | --- | --- | --- |
| 1 | 3 | 3 | 3 | 6 | 0 |
| 2 | 6 | 3 | 6 | 9 | 50 |
| 3 | 9 | 3 | 9 | 12 | 66.7 |
| 4 | 12 | 6 | 12 | 18 | 50 |
| 5 | 15 | 6 | 15 | 21 | 60 |
| 6 | 18 | 6 | 18 | 24 | 66.7 |
| 7 | 21 | 9 | 21 | 30 | 57.1 |
| 8 | 24 | 9 | 24 | 33 | 62.5 |
| 9 | 27 | 9 | 27 | 36 | 66.7 |
| 10 | 30 | 12 | 30 | 42 | 60 |

**Table S3.1.** Partial table of results. The number (n) of consultants in the second column is treble the n of theatres as per previous modelling^18^ which is also equivalent to the n of AAs in a 1:2 model (column 4). For the n of consultants in a 1:3 model (column 3) is 1/3^rd^ the number of theatres (with the next even n of theatres used for odd numbers of theatres), rounded up. The total number of staff is the sum of columns 3 and 4. The last column is the maximum economically viable salary of an AA. This is calculated as the n of AAs needed (column 4) divided by the n of consultant posts theoretically saved (the difference between columns 2 and 3), expressed as a %. Note the sawtooth or oscillatory nature of the result.

Figure S3.1 shows these results plotted along with the results of the primary modelling for 1:2 ratios (see Figure 2, main text) and also for less leave for AAs (see Online Supplement S2). The 1:3 ratio permits a higher AA salary as fewer consultant posts are needed, and the combination of leave allocations with supervision ratios can be adjusted to yield an optimum balance, if required.

**Figure S2.2.** Green line: the maximum salary that can be awarded to an AA, as a proportion of their supervisor’s salary, assuming a 1:3 supervision ratio per operating theatre (allocated 2 sessions over 5 days of the week). The ‘sawtooth’ pattern arises as a result of the ‘staircase’ relationship in Figure 1, itself a function of the absences due to leave and non-clinical time within contracts. The black line (reproduced from Figure 2 in the main paper) assumes that AAs have the same leave and non-clinical time entitlements as their supervisors. The red line assumes that they have half this entitlement.


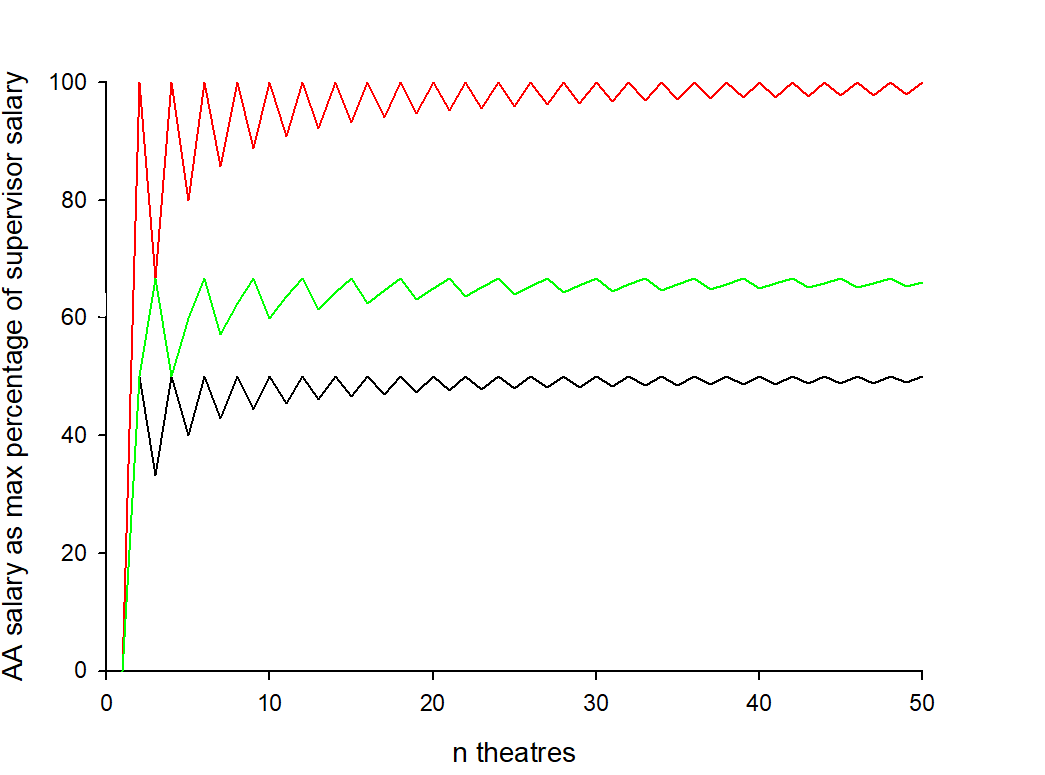

Supplement: Multimedia component 1 [file mmc1.docx]
